# A Simple Tribute to My Devoted Friend of the Deathless Dead

**Published:** 1914-02

**Authors:** A. W. Fly


### A Simple Tribute to my Devoted Friend of the Deathless Dead.

When Marcellus E. Kleberg departed this life his family, his kindred and his friends lost the loveliest nature I have ever known. A companion, the loss of which can never be filled. He was the greatest Christian that I ever met without a creed; a churchman without a house of worship; the greatest Democrat I have ever seen. He believed in the democracy of the nation, the democracy of the home, the fireside, the democracy of the soul, and even the democracy of thought.

The Sermon upon the Mount, the Declaration of the American Independence, the Constitution of the United States and the Golden Rule were the basic guides of his life's action. He was as free from guile as a new-born babe; he never sacrificed principle for policy; prejudice had no part in his makeup; charity, sincerity and loyalty to whatever he conceived to be right were the guiding stars that lighted his pathway from the cradle to the grave.

I have listened to many of the great orators—to my mind I never heard one who, according to my conception, so completely filled to the full measure the definition of that term. His absolute consciousness in the rectitude of the position he took seemed to be like an electric storm, laying dormant to be touched off by some inspiration directed in its motive as an excitant power.

Many of his great speeches were published; the greatest two were not published. One when he responded to the toast, "To the President of the United States," during the first visit of the battleship Texas. I was toastmaster, and when I thought he was sufficiently keyed to action, I called upon him to respond to the above toast. While the beginning of the flight of oratory, someone mentioned that it was Washington's birthday; that touched the electric button, and for twenty minutes eloquence flowed from his lips like a mighty torrent of matchless melodies; his eyes flashed the lightening spark that was but his outward expressions of his innermost soul; he seemed inspired with the idea of personal liberty and what Presidents of the United States meant to the great American people, and his diction illustrated that great Biblical expression:

"From the abundance of the heart the mouth speaketh."

The other was at the grave of my old negro servant [The "Old Secretary"]. For genuine pathos and affectionate expression for the lowly who was there buried, I do not believe that a greater art of the genuine pathos could have been expressed by man. In the closing words, "God bless the old 'Secretary,'" there was not a dry eye on the ground.

In conclusion, I will say, the family, kindred and his friends, except for the loss of his companionship, should not feel one iota of regret, because he lived to nearly three score and ten; his life was an open book and could be read by all men, without one blot on his wonderful manhood.

I am here reminded of the beautiful lines of Savage:

"New being is from being ceased;
No life is but by death;
Something expiring everywhere to give some other breath.

There is not a flower that glads the spring
But blooms upon the grave of its dead parent seed,
O'er which its forms of beauty wave.
The oak, that like an ancient tower stands massive on the heath,
Looks out upon a living world but strikes its roots in death.

Today is but a structure built upon dead yesterday
And progress hews her temple stones from wrecks of old decay.

Then mourn not: death: 'tis but a stair
Built with divinest art upon which the deathless footsteps climb
Of loved ones who depart."

A. W. Fly, M. D.

